# Zinc induces CDK5 activation and neuronal death through CDK5-Tyr15 phosphorylation in ischemic stroke

**DOI:** 10.1038/s41419-018-0929-7

**Published:** 2018-08-29

**Authors:** Qing-Zhang Tuo, Zhen-Yu Liuyang, Peng Lei, Xiong Yan, Yang-Ping Shentu, Jia-Wei Liang, Huan Zhou, Lei Pei, Yan Xiong, Tong-Yao Hou, Xin-Wen Zhou, Qun Wang, Jian-Zhi Wang, Xiao-Chuan Wang, Rong Liu

**Affiliations:** 1 Department of Pathophysiology, Key Laboratory of Ministry of Education for Neurological Disorders, School of Basic Medicine, Tongji Medical College, Huazhong University of Science and Technology, Wuhan, China; 2 Department of Neurology and State Key Laboratory of Biotherapy, West China Hospital, Sichuan University, and Collaborative Innovation Center for Biotherapy, Sichuan, China; 3 The Institute for Brain Research, Collaborative Innovation Center for Brain Science, Huazhong University of Science and Technology, Wuhan, China

## Abstract

CDK5 activation promotes ischemic neuronal death in stroke, with the recognized activation mechanism being calpain-dependent p35 cleavage to p25. Here we reported that CDK5-Tyr15 phosphorylation by zinc induced CDK5 activation in brain ischemic injury. CDK5 activation and CDK5-Tyr15 phosphorylation were observed in the hippocampus of the rats that had been subjected to middle cerebral artery occlusion, both of which were reversed by pretreatment with zinc chelator; while p35 cleavage and calpain activation in ischemia were not reversed. Zinc incubation resulted in CDK5-Tyr15 phosphorylation and CDK5 activation, without increasing p35 cleavage in cultured cells. Site mutation experiment confirmed that zinc-induced CDK5 activation was dependent on Tyr15 phosphorylation. Further exploration showed that Src kinase contributed to zinc-induced Tyr15 phosphorylation and CDK5 activation. Src kinase inhibition or expression of an unphosphorylable mutant Y15F-CDK5 abolished Tyr15 phosphorylation, prevented CDK5 activation and protected hippocampal neurons from ischemic insult in rats. We conclude that zinc-induced CDK5-Tyr15 phosphorylation underlies CDK5 activation and promotes ischemic neuronal death in stroke.

## Introduction

Acute ischemic stroke is the most common type of stroke and occurs as a result of vascular occlusion^1^. Ischemic brain injury develops as the result of ischemia/reperfusion with multiple mechanisms involved including inflammation, excitotoxicity, oxidative stress and apoptosis^2^. Zinc is a trace element, which is enriched in the brain, with crucial functions in the physiology and pathophysiology of the central nerves system^3^. In ischemic brain injury, increased Zn^2+^ in cytoplasm, which were originated from extracellular influx and intracellular release from metallothioneins (MTs) or organelles play a key role in promoting ischemic neuronal damage, leading to necrotic, apoptotic and autophagic cell death^4^. Zn^2+^ accumulation precedes calcium deregulation in ischemia-damaged neurons; uptake of Zn^2+^ by mitochondria is associated with mitochondrial depolarization and consequent Ca^2+^ deregulation^5^. Zn^2+^ also acts on a selective zinc-sensing receptor ZnR to induce intracellular release of Ca^2+^^6^. Thus, excitotoxic [Zn^2+^]_i_ rise is an early event before Ca^2+^ deregulation in the promotion of neuronal death. However, the underlying molecular signaling of Zn^2+^-induced neuronal death has not been fully elucidated.

CDK5 is a serine–threonine kinase, which is structurally similar to the mitotic cyclin-dependent kinases^7^. CDK5 shows neuron-specific activity because its activation requires association with the neuron-specific activator p35 or p25, the truncated form of p35 by the cleavage of calpain^8,9^. CDK5 activity is also regulated by phosphorylation at Tyr15, which induces CDK5 activation^10–12^. Abnormal CDK5 activity has been reported to contribute to pathogenesis of several neurological diseases such as Alzheimer’s disease^13,14^, Parkinson’s disease^15^, amyotrophic lateral sclerosis (ALS)^16^ and stroke^17,18^. In a transient forebrain ischemic rat model, CDK5 was activated specifically in hippocampal CA1 region and induced cell death through phosphorylating N-methyl-D-aspartic acid (NMDA) receptors, with the other regions of the hippocampus uninfluenced^19^. At the same time, chelatable zinc also accumulated specifically in degenerating neurons in the hippocampal CA1 and other presynaptic zinc-containing brain regions, preceding neurodegeneration^20^. These facts raise the possibility that zinc may play a role in CDK5 regulation in ischemic brain injury.

Here we explore the regulatory effect of zinc on CDK5 in cultured cells and in animal models with middle cerebral artery occlusion (MCAO). The results show that CDK5 activation was accompanied with Tyr15 phosphorylation in the hippocampus of the rats that had been subjected to MCAO, both of which were reversed by pretreatment with zinc chelator; whereas p35 cleavage and calpain activation in ischemia were not reversed. Zinc induced CDK5 activation through Src kinase-dependent Tyr15 phosphorylation in ZnSO_4_-incubated cells. Src kinase inhibition or expression of an unphosphorylable mutant Y15F of CDK5 abolished Tyr15 phosphorylation, prevented CDK5 activation and protected hippocampal neurons from ischemic insult. Our data suggest that zinc-induced CDK5-Tyr15 phosphorylation promotes CDK5 activation and the following ischemic neuronal death in stroke.

## Materials and methods

### C57Bl/6 mice and Sprague-Dawley rats

Adult (3 months old) male Sprague-Dawley (SD) rats weighing 250–300 g and adult (3 months old) male C57Bl/6 mice weighing 18–22 g were housed individually under standard conditions of temperature and humidity, and a 12-h light/dark cycle (lights on at 08:00), with free access to food and water before use. Adequate measures were taken to minimize pain or discomfort during surgeries. All animal experiments were approved by the Animal Care and Use Committee of Huazhong University of Science and Technology, and performed in compliance with the National Institutes of Health Guide for the Care and Use of Laboratory Animals.

### Establishment of MCAO model

Transient acute focal cerebral ischemia was induced by intraluminal MCAO, as described previously^21^. Briefly, after weighing, animals were deeply anesthetized. A 4-mm distal nylon monofilament (30 mm in length, 0.16 mm in diameter, Amber, Japan) segment was coated with 0.21–0.22 mm diameter silicone (Henkel, Australia) for mice, and an 11-mm distal monofilament (50 mm in length, 0.23 mm in diameter) segment was coated with 0.28–0.30 mm diameter silicone for rats. MCAO was performed by inserting the monofilament via the common carotid artery into the left internal carotid artery, advanced 9–10 mm (in mice) or 20–21 mm (in rats) past the carotid bifurcation until a slight resistance was felt. Body temperature and respiratory rate were monitored during surgery. Body temperature of the animal was maintained at 37 ± 0.5 °C throughout the procedure using a heat pad. For inducing MCAO, the filament was left in place for 60 min, and then withdrawn for reperfusion. In the sham group, the filament was inserted only 5 mm above the carotid bifurcation. Mice were excluded from further studies if excessive bleeding occurred during surgery, if the operation time exceeded 90 min, or if hemorrhage was found in the brain slices during postmortem examination.

### Drug treatments

For rat clioquinol (CQ) treatment, one dose of CQ (10 mg/kg) or vehicle (Dmethyl sulfoxide, DMSO) was injected into the SD rats intraperitoneally (i.p.) 60 min before surgery. For rat 4-Amino-3-(4-chlorophenyl)-1-(t-butyl)-1H-pyrazolo[3,4-d]pyrimidine (PP2) treatment, one dose of PP2 (10 mM, 10 μl) or vehicle (DMSO) was injected into the SD rats intracerebroventricularly (i.c.v.) 60 min before surgery. For treatment on cultured cells, the final concentration of PP2 was 1 μM.

### TTC staining

After 24 h of MCAO/reperfusion, mice and rats were weighed and sacrificed. The brain was removed rapidly and frozen at –20 °C for 20 min. Coronal slices were made at 2 mm intervals from the frontal poles. Sections were immersed in 1% 2,3,5-tripenyltetrazolium chloride (TTC, T8877, Sigma-Aldrich, USA) in phosphate-buffered saline (PBS) at 37 °C for 20 min. The presence or absence of infarction was determined by examining TTC-stained sections for the areas on the side of infarction that did not stain with TTC.

### Quantitative measurement of brain infarct volume

Serial sections were photographed using a digital camera and the area of infarct was quantified with Image J (NIH, Bethesda, MD, USA). The area of infarct (white, unstained), the area of ipsilateral hemisphere (white, unstained, plus red brick, stained) and the area of the contralateral hemisphere (red brick, stained) were measured for each section by an investigator blinded to the experimental group. The volume was calculated by summing the representative areas in all sections and multiplying by the slice thickness, then correcting for edema, as previously described:^21^ Corrected infarct volume = contralateral hemisphere volume – (ipsilateral hemisphere volume – infarct volume).

### Nissl staining

The rats and mice were deeply anesthetized and then fixed by trans-cardial perfusion with 0.9% NaCl, followed by 4% paraformaldehyde in 100 mM phosphate buffer. After perfusion, the brains were post-fixed in the same solution for 2 days at 4 ℃, followed with dehydration in 30% saccharose for 9 days at 4 °C. Then, the samples were embedded in optimum cutting temperature compound (OCT, Sakura, USA), frozen and sectioned at 30 μm using freezing microtome (Leica 1950, Wetzlar, Germany). Coronal sections of the brain were immersed in 1% toluidine blue for 3 min, followed by dehydration in 75, 85, 95 and 100% ethanol solutions, transparented using xylene, placed under cover slips and observed under microscope (SV120, Olympus Corporation, Japan). The neuron numbers per ×40 magnification in the CA1 area were quantified by an investigator blinded to the experimental group, using Imaging-Pro Plus 6.0 (Media Cybernetics Inc., MD, USA).

### Cloning and generation of plasmids and AAV virus particles

Mutant Y15F-CDK5 was generated from wild-type mouse CDK5 through mutagenesis by company (OBIO, Shanghai, China). The sequences of wild-type CDK5 or Y15F-CDK5 were then inserted to a pcDNA3.1-GFP vector to generate pcDNA3.1-GFP-CDK5 and pcDNA3.1-GFP-Y15F-CDK5 plasmids. The plasmids were transfect into N2a cells with Lipofectamine 2000 (Invitrogen Corporation, CA, USA) to induce CDK5 overexpression. Adeno-associated viruses (AAVs) pAAV-CMV-CDK5-EGFP and pAAV-CMV-Y15F-CDK5-EGFP expressing wild-type or Y15F mutated CDK5 were constructed and packaged in OBIO (Shanghai, China). Virus particles were infused (1.0 μl at 0.1 μl/min) into the CA1 of the right hippocampus.

### Sample preparation and western blotting

Rats or mice were deeply anesthetized and trans-cardial perfusion with PBS was performed before the brains were removed. Affected hippocampus tissues were homogenized in ice-cold RIPA buffer containing 50 mM Tris (pH 7.4), 150 mM NaCl, 1% NP-40, 0.25% sodium deoxycholate, 1 mM EDTA, 1 mM sodium orthavanadium, 100 μM phenylmethylsulfonyl fluoride (PMSF). Total protein concentration was determined by BCA protein assay (Thermo Fisher Scientific, USA). Aliquots of homogenate with equal protein concentration were separated by 10% (v/v) sodium dodecyl sulfate/polyacrylamide gel electrophoresis (SDS/PAGE) gel, and then transferred to nitrocellulose membrane (Amersham Biosciences, Pittsburgh, USA). After blocking in 5% (w/v) nonfat milk for 30 min at room temperature, the membranes were then incubated with primary antibodies at 4 °C overnight, followed by incubation with anti-goat, anti-rabbit or anti-mouse IgG conjugated to IRDye for 1 h at room temperature and visualized using the Odyssey Infrared Imaging System (Licor Biosciences, Lincoln, NE, USA). The primary antibodies used were as follows: CDK5 (1:500, sc-6247, Santa Cruz Biotechnology), phospo-CDK5 at Tyr15 (1:500, sc-12918, Santa Cruz Biotechnology), p35 (1:1000, sc-820, Santa Cruz Biotechnology), p25 (1:500, AP060, Beyotime, China), Src (1:100, 05-184, Millpore), Fyn (1:1000, sc-434, Santa Cruz Biotechnology), phospo-Src at Tyr 529 (1:500, S2065, Sigma-Aldrich), phospo-SFK at Tyr 416 (1:1000, 2101S, Cell Signaling Technology) and DM1A (1:1000, T9026, Sigma-Aldrich).

### CDK5 and calpain activity assay

To determine CDK5 activity, brain tissue or cell samples were homogenized in ice-cold lysis buffer containing 50 mM tris-HCl (pH 7.6), 150 mM NaCl, 1% NP-40, 2 mM EDTA, 1 mM sodium orthovanadate and proteinase inhibitor cocktail (Sigma, 1:1000). After clearing debris by centrifuging at 12,000 rpm at 4 °C, protein concentrations in the extracts were determined using the BCA protein assay. The extracts (100 μg protein) were incubated with monoclonal mouse anti-CDK5 (sc-6247, 1 μg, Santa Cruz Biotechnology, for endogenous CDK5 activity assay) or monoclonal mouse anti-GFP (ab1218, 1 μg, Abcam, for exogenously expressed CDK5 activity assay) overnight at 4 °C, followed by the addition of 20 μl of Protein A + G agarose (CW0349S, CWbiotech, China) for 3 h at 4 °C. Immunoprecipitates were washed four times with lysis buffer, and beads were then resuspended in kinase buffer, containing 20 mM Tris-HCl (pH 7.6), 20 mM MgCl_2_, 2 mM MnCl_2_, 1 mM EDTA, 1 mM EDTA, 0.1 mM dithiothreitol, 2 μl ATP (20-306, Millipore) and Histone-H1 (5 mg/ml,14-155, Millipore). Samples were subjected to 10% (v/v) SDS/PAGE gel for western blotting. The antibodies used for samples were: CDK5 (1:500, sc-6247, Santa Cruz Biotechnology), Histone-H1 (1:500, 05-457, Millpore), phospo-Histone-H1(1:500, 05-1324, Millpore). Calpain activity was measured by ELISA following the manufacturer's instruction through using a calpain activity assay kit (ab65308, Abcam, Cambridge, MA).

### Immunofluorescence

Mice brain tissues were fixed and sliced as that in Nissl staining, sections were permeabilized in 0.3% Triton X-100 for 30 min, followed by incubation with 5% normal goat serum for 30 min to block nonspecific sites at room temperature. Primary antibody incubation (NeuN, 1:200, MAB377, Millpore) was performed for 48 h at 4 °C. Alexa Fluor 546-conjugated secondary antibody (1:200) was used for fluorescence labeling. 4,6-Diamidino-2-phenylindole (DAPI) was used to label the nuclear. The image was observed with the LSM780 confocal microscope (Carl Zeiss, Oberkochen, Germany).

### Statistical analysis

Data were expressed as mean ± SEM and analyzed using SPSS 20.0 statistical software (SPSS Inc., Chicago, IL, USA). The one-way analysis of variance (ANOVA) procedure followed by Least Significant Difference (LSD) post hoc test was used to determine the differences among groups. For data from two groups, two-tailed Student’s *t*-test was used. The significance was set at *p* *<* 0.05. All results shown correspond to individual representative experiments.

## Results

### CDK5 is activated with Tyr15 phosphorylation and p35 cleavage in ischemic injury

**Fig. 1:**
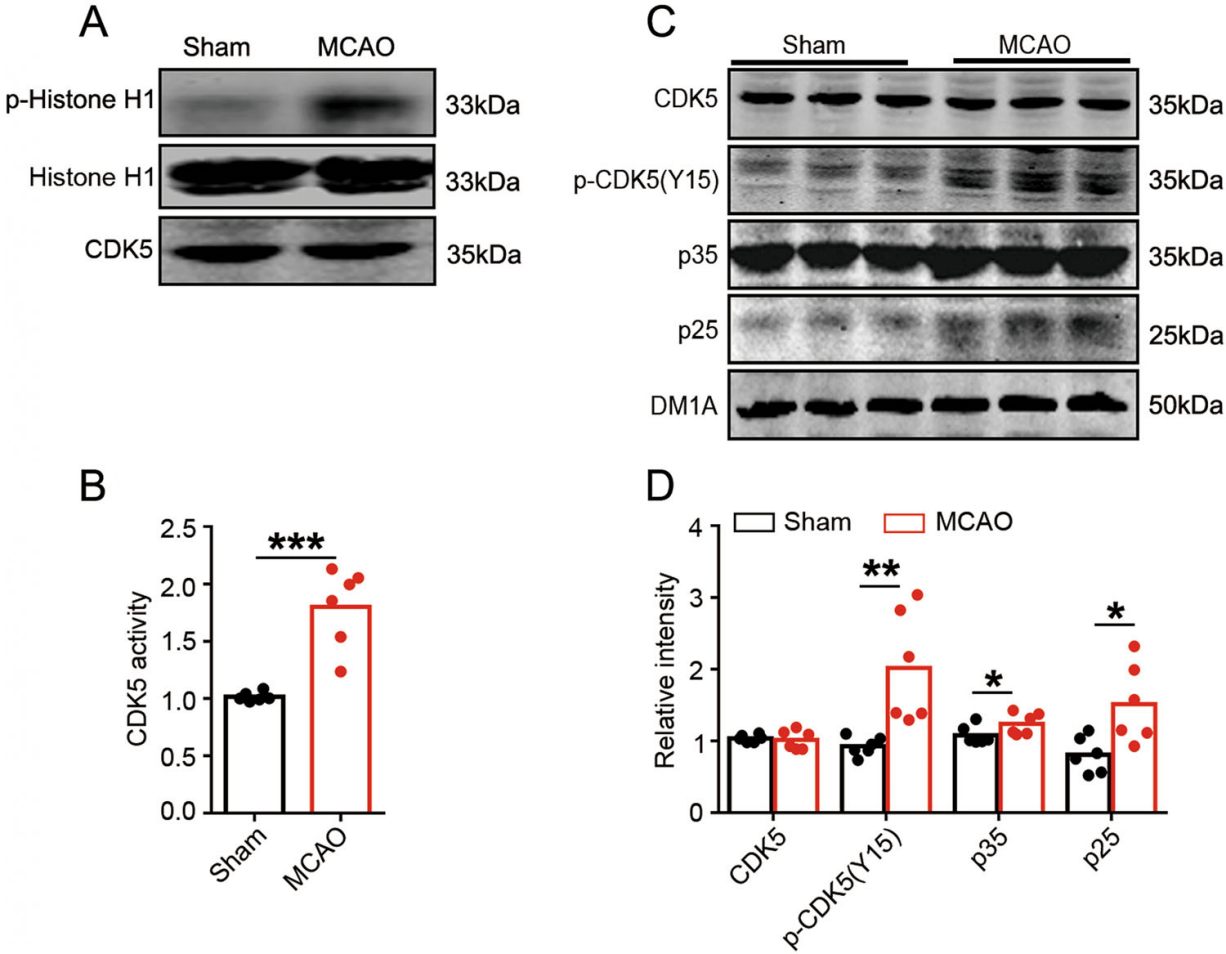
CDK5 is activated with Tyr15 phosphorylation and p35 cleavage in ischemic injury. **a** CDK5 is activated in hippocampus of MCAO rats. Brain tissue homogenates were prepared from rat hippocampus of Sham and MCAO rats (artery occlusion for 60 min, followed with 24-h reperfusion). CDK5 activities were detected by incubating the immunoprecipitated CDK5 from the homogenates with its substrate Histone-H1 and ATP. At the end of incubation, the levels of phosphorylated and total Histone-H1 were shown by western blotting. **b** CDK5 activities were evaluated by calculating the ratio of phosphorylated to total Histone-H1 levels in **a** (*n* = 6 animals per group, Sham vs MCAO, *t*-test, *p* *=* 2.673E-05, ***p < 0.001). **c** Phosphorylation of CDK5 at Tyr15 and cleavage of p35 to p25 are increased in hippocampus of MCAO rats. Levels of total and Tyr15 phosphorylated CDK5, p35 and p25 in brain homogenates were detected by using the indicated antibodies. **d** Quantitative analysis of the protein levels in **c** (*n* = 6 animals per group, *t*-test, from left to right, *p* *=* 0.0034, *p* *=* 0.0015, *p* *=* 0.0149, *p < 0.05, **p < 0.01)

To explore the mechanism underlying CDK5 activation in brain ischemic injury, we established an animal model in which focal ischemia is induced by selective unilateral occlusion of the MCAO in rats. Reperfusion is achieved after 1 h of MCAO by removing the embolism. Unilateral MCAO caused marked CDK5 activation at 6 h after reperfusion (Figs. 1a, b), with a significant increase of Tyr15 phosphorylation of CDK5 (Figs. 1c, d). Increased level of p25 was observed, indicating p35 cleavage. P35 also showed a slight increase, possible due to a compensatory overexpression during ischemia (Figs. 1c, d). Thus, CDK5 is activated in focal cerebral ischemic injury, accompanied with CDK5-Tyr15 phosphorylation and p35 cleavage.

### Zinc chelator CQ reverses ischemic injury, CDK5 activating and Tyr15 phosphorylation, but not p35 cleavage

**Fig. 2:**
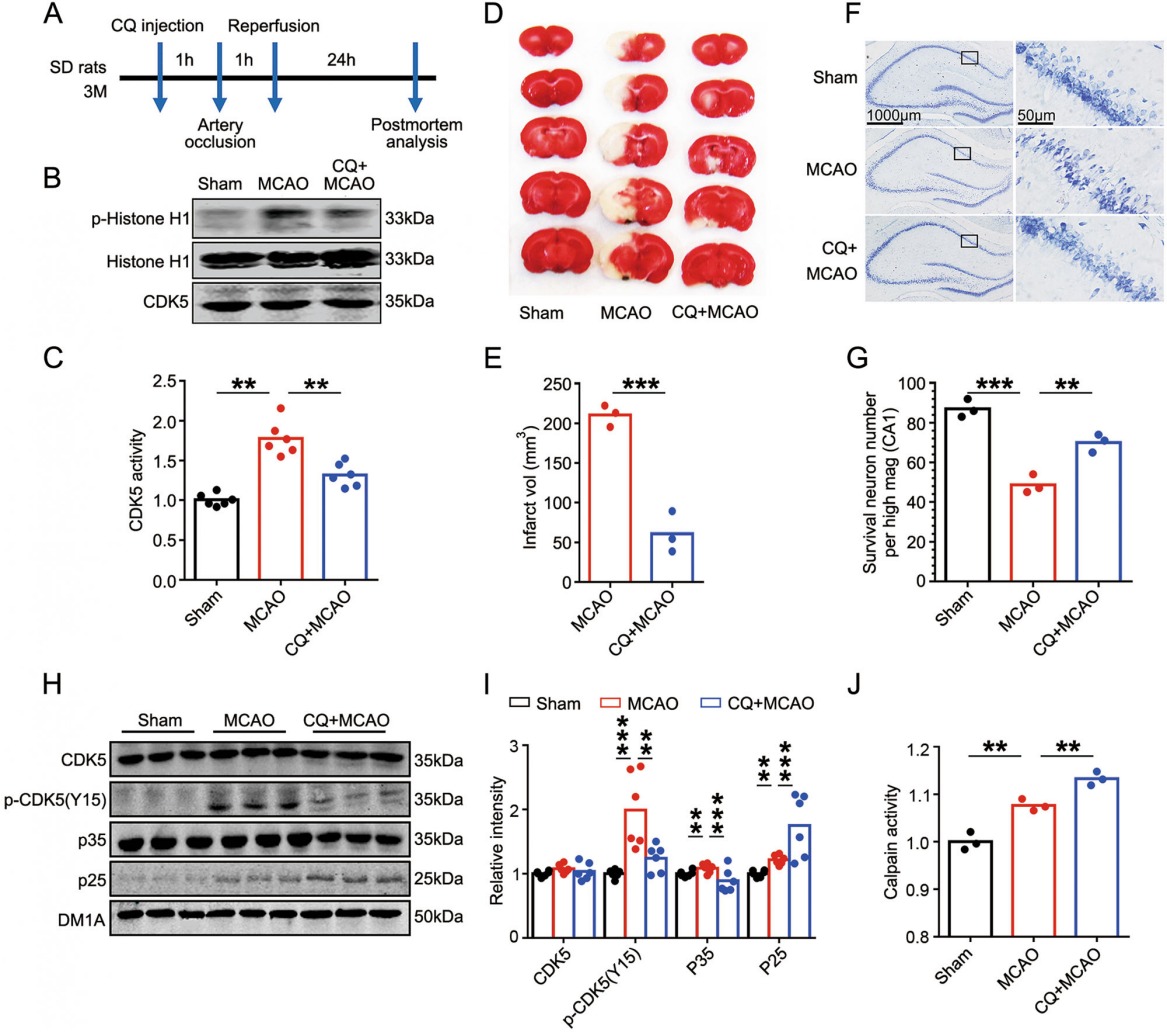
Zinc chelator reverses ischemic injury, CDK5 activating and Tyr15 phosphorylation, but not p35 cleavage in MCAO rats. **a** Timeline of the experiment. One dose of zinc chelator CQ (10 mg/kg) or vehicle (DMSO) was injected into the SD rats intraperitoneally (i.p.) 60 min before MCAO. **b** Ischemia-induced CDK5 activating was reversed by CQ pretreatment. CDK5 activities were detected by evaluating the phosphorylation level of Histone-H1. Representative blots with antibodies as indicated. **c** CDK5 activities were evaluated by calculating the ratio of phosphorylated to total Histone-H1 levels in **b** (*n* = 6 animals per group, one-way ANOVA followed by LSD’s post hoc tests, from left to right, *p* *=* 0.006, *p* *=* 0.005, **p < 0.01). **d** Infarct size in MCAO rat brain was reduced by the pretreatment of zinc chelator CQ. Representative TTC-stained serial brain sections were shown from sham, MCAO and CQ + MCAO rats. **e** Quantification of infarct volume (vol) indicated by TTC staining (*n* = 3 animals per group, one-way ANOVA followed by LSD’s post hoc tests, *p* *=* 0.0009, ***p < 0.001). **f** CQ reduced CA1 pyramidal cell death induced by ischemia. Representative Nissl staining image of the hippocampus sections from sham, MCAO and CQ + MCAO rats. **g** Quantification of survival neuron numbers indicated by Nissl staining (*n* = 3 animals per group, one-way ANOVA followed by LSD’s post hoc tests, from left to right, *p* *=* 0.0006, *p* *=* 0.0019, **p < 0.01,***p < 0.001). **h** Ischemia-induced phosphorylation of CDK5 at Tyr15 was reversed by CQ pretreatment. Levels of total and Tyr15 phosphorylated CDK5, p35 and p25 in brain homogenates were detected by using the indicated antibodies. **i** Quantitative analysis of the protein levels in **h** (*n* = 6 animals per group, one-way ANOVA followed by LSD’s post hoc tests, from left to right, *p* *=* 0.0006, *p* *=* 0.0025, *p* *=* 0.0043, *p* *=* 4.1839E-05, *p* *=* 0.0024, *p* *=* 4.40967E-05, ***p < 0.001, **p < 0.01). **j** Calpain activity was further enhanced by CQ pretreatment. Activities of calpain were analyzed in the three groups by ELISA assay (*n* = 6 animals per group, one-way ANOVA followed by LSD’s post hoc tests, from left to right, *p* *=* 0.0042, *p* *=* 0.0071, **p < 0.01)

CQ is able to reduce chelatable zinc in brain^22^ and attenuate the ischemia-induced zinc accumulation in the CA1 pyramidal neurons^23^, thus we used CQ to reveal the effect of zinc on CDK5 activation in focal cerebral ischemia (Fig. 2a). Intraperitoneal injection of CQ 1 h before MCAO showed no significant effect on the cerebral blood flow assayed by laser Doppler flow immediately after the occlusions (Supplementary Fig 1).

However, CQ abolished CDK5 activation (Figs. 2b, c), significantly reduced the infarct volume detected by TTC staining (Figs. 2d, e) and neuronal loss in hippocampal CA1 detected by Nissl staining (Figs. 2f, g). Meanwhile, CDK5-Tyr15 phosphorylation was reversed (Figs. 2h, i). CQ pretreatment did not prevent the cleavage of p35 to p25. On the contrary, p35 cleavage was enhanced, as indicated by decreased p35 and increased p25 level (Figs. 2h, i). To confirm the increased p35 cleavage in CQ group, the calpain activities were detected. The results showed that MCAO induced calpain activation, CQ pretreatment further enhanced calpain activity (Fig. 2j), which can explain the increased p35 cleavage in this group. Thus, zinc chelator rescues ischemic injury, CDK5 activating and Tyr15 phosphorylation, but not p35 cleavage. Tyr15 phosphorylation of CDK5 is possibly the upstream factor of CDK5 activation in cerebral ischemic injury, and endogenously motivated zinc may promote this effect.

### Zinc induces CDK5 activation through Tyr15 phosphorylation but not p35 cleavage

**Fig. 3:**
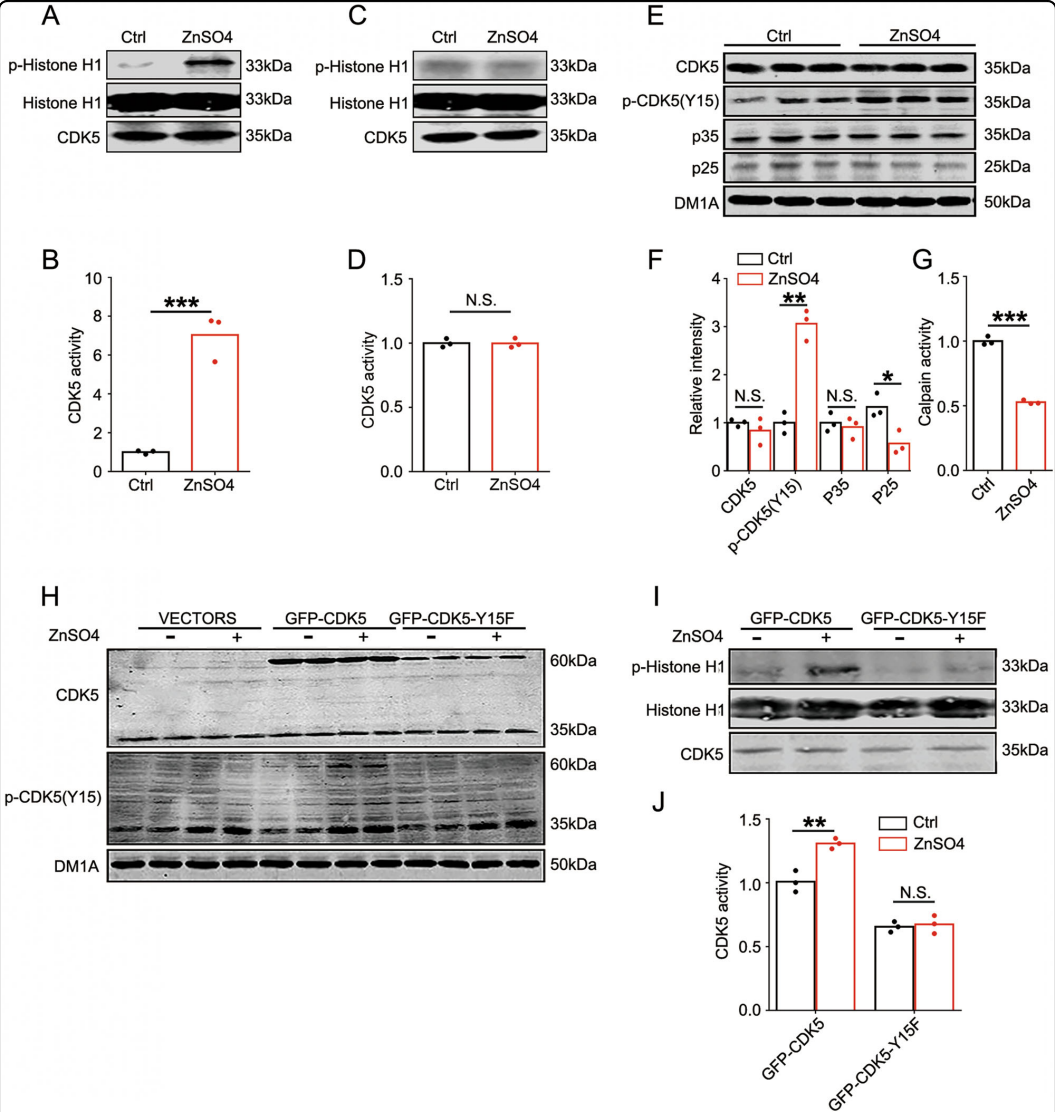
Zinc induces CDK5 activation through Tyr15 phosphorylation. **a** Zinc activated CDK5 in N2a cells. CDK5 activities were measured in cell lysates prepared from N2a cell incubated with or without ZnSO_4_ (10 μM) for 3 h by evaluating the phosphorylation level of Histone-H1. Representative blots with antibodies as indicated. **b** CDK5 activities were evaluated by calculating the ratio of phosphorylated to total Histone H1 levels in **a** (*n* = 3 cell experiment per group, *t*-test, *p* *=* 0.0009, ***p < 0.001). **c** Activation of CDK5 by zinc required the cellular signaling transduction. N2a cell lysates were incubated with or without ZnSO_4_ (10 μM) for 3 h, the direct effect of zinc on CDK5 activities were observed. **d** Quantitative analysis of CDK5 activities in **c** (*n* = 3 tube experiment per group, *t*-test, *p* *=* 0.35). **e** Zinc-induced CDK5 phosphorylation at Tyr15. Levels of total and Tyr15 phosphorylated CDK5, p35 and p25 in N2a cell lysates in **a** were detected by using the indicated antibodies. **f** Quantitative analysis of the protein levels in **e** (*n* = 3 cell experiment per group, *t*-test, from left to right, *p* *=* 0.0008, *p* *=* 0.0201, **p < 0.01, *p < 0.05). **g** Zinc inhibited calpain activity in N2a cells. Activities of calpain in the two groups were analyzed by ELISA assay (*n* = 3 cell experiment per group, *t*-test, *p* *=* 2.452E-05, ***p < 0.001). **h** GFP-tagged wild-type CDK5 (GFP-CDK5) or Y15F mutated CDK5 (GFP-CDK5-Y15F) were overexpressed in N2a cells with or without ZnSO_4_ (10 μM, 3 h) incubation. The expression of exogenous CDK5 and its phosphorylation at Tyr15 were evaluated by western blotting using the antibodies indicated. **i** Exogenously expressed CDK5 was immunoprecipitated with antibody against GFP and the CDK5 activities were detected by evaluating the phosphorylation level of Histone-H1. Representative blots with antibodies as indicated. **j** CDK5 activities were evaluated by calculating the ratio of phosphorylated to total Histone-H1 levels in **i** (*n* = 3 cell experiment per group, *t*-test, from left to right, *p* *=* 0.0051, *p* *=* 0.7020, **p < 0.01)

To confirm the effect of zinc on Tyr15 phosphorylation and activation of CDK5, N2a cells were incubated with zinc sulfate directly. Incubation of intact cells with ZnSO_4_ for 3 h resulted in CDK5 activating as indicated by CDK5 activity assay (Figs. 3a, b). Incubation of N2a cell lysate with ZnSO_4_ did not increase CDK5 activity (Figs. 3c, d), indicating that CDK5 activation is not induced by a direct effect of zinc ions on the enzyme, but dependent on signaling transduction in intact cells. Further detection showed that zinc treatment induced Tyr15 phosphorylation of CDK5 (Figs. 3e, f). Zinc incubation did not increase the p35 cleavage to p25; on the contrary, p25 level was decreased (Figs. 3e, f). Consistent with this finding, calpain activity was downregulated in zinc-treated cells (Fig. 3g), indicating a reduced p35-p25 cleavage. These data indicated that zinc may activate CDK5 through Tyr15 phosphorylation but not p35/p25 signaling. To further confirm this hypothesis, a non-phosphorylable CDK5 mutant Y15F was overexpressed in N2a cells, which were subjected to ZnSO_4_ incubation (Fig. 3h). Compared with wild-type CDK5, Y15F mutation resulted in decreased enzyme activity, and zinc showed no effect on Y15F-CDK5 activation (Figs. 3i, j). These data indicate that zinc induces CDK5 activation through Tyr15 phosphorylation but not calpain-dependent p35 cleavage.

### Zinc induces CDK5-Tyr15 phosphorylation through Src activation

**Fig. 4:**
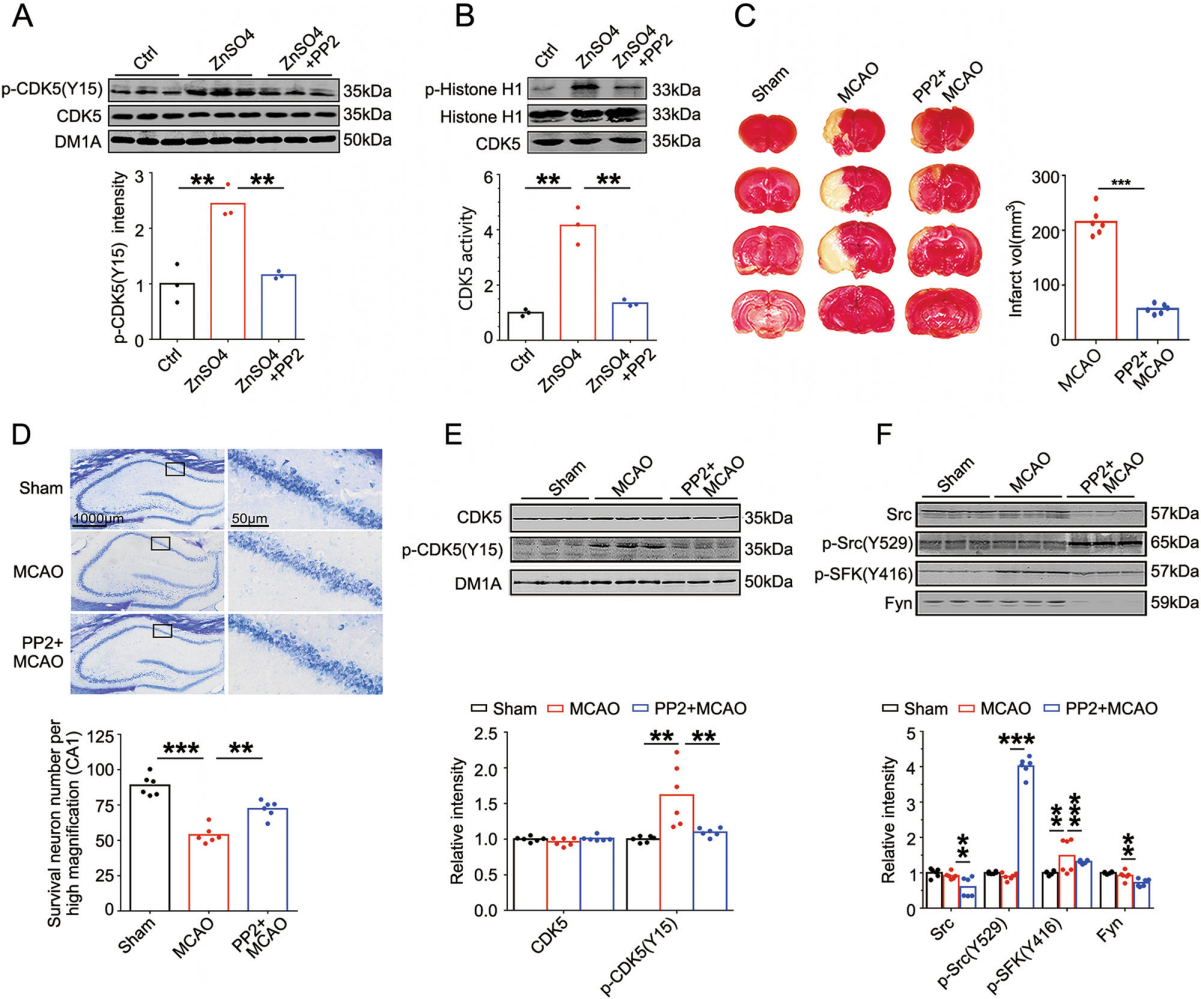
Zinc induces CDK5-Tyr15 phosphorylation through Src activation. **a** Src inhibitor PP2 reversed zinc-induced CDK5-Tyr15 phosphorylations in N2a cells. Upper, levels of total and Tyr15 phosphorylated CDK5 were detected in N2a cell lysates prepared from Ctrl, ZnSO_4_ (10 μM, 3 h) and ZnSO_4_ + PP2 (PP2 in 1 μM pretreated at 1 h before zinc incubation) by using the indicated antibodies. Bottom, quantitative analysis of the levels of Tyr15- phosphorylated CDK5 (*n* = 3 cell experiment per group, one-way ANOVA followed by LSD’s post hoc tests, from left to right, *p* *=* 0.0056, *p* *=* 0.0019, **p < 0.01). **b** Src inhibitor PP2 reversed zinc-induced CDK5 activation in N2a cells. Upper, CDK5 activities in cell lysates in **a** were detected by evaluating the phosphorylation level of Histone-H1. Representative blots with antibodies as indicated. Bottom, quantitative analysis of the CDK5 activities in three groups (*n* = 3 cell experiment per group, one-way ANOVA followed by LSD’s post hoc tests, from left to right, *p* *=* 0.0013, *p* *=* 0.0021, **p < 0.01). **c** Src inhibitor PP2 reduced the infarct size in MCAO rats. Left, representative TTC-stained serial brain sections were shown from sham, MCAO (at 24-h reperfusion) and PP2 + MCAO (PP2 10 mM 10 μl, i.c.v., at 1 h before ischemia) rats. Right, quantification of infarct volume (vol) indicated by TTC staining (*n* = 6 animals per group, *t*-test, *p* *=* 0.0005, ***p < 0.001). **d** Src inhibitor PP2 reduced CA1 pyramidal cell death induced by ischemia. Upper, representative Nissl staining image of the hippocampus sections from sham, MCAO and PP2 + MCAO rats. Bottom, quantification of survival neuron numbers indicated by Nissl staining (*n* = 6 animals per group, one-way ANOVA followed by LSD’s post hoc tests, from left to right, *p* *=* 0.0005, *p* *=* 0.0030, **p *<* 0.01, ***p < 0.001). **e** Src inhibitor PP2 reversed Tyr15 phosphorylation of CDK5 in MCAO rats. Upper, brain tissue homogenates were prepared from rat hippocampus of Sham, MCAO and PP2 + MCAO rats. Levels of total and Tyr15 phosphorylated CDK5 in brain homogenates were detected by using the indicated antibodies. Bottom, quantitative analysis of the protein levels (*n* = 6 animals per group, one-way ANOVA followed by LSD’s post hoc tests, from left to right in p-CDK5(Y15) level analysis, *p* *=* 0.0024, *p* *=* 0.0042, **p < 0.01). **f** PP2 reversed Src activation in MCAO rats. Upper, total Src kinases (Src and Fyn), active form (Y416) and inactive form (Y529) of Src kinases in **e** were detected by western blotting. Bottom, quantitative analysis of the protein levels as indicated (*n* = 6 animals per group, one-way ANOVA followed by LSD’s post hoc tests, from left to right, *p* *=* 0.0014, *p* *=* 7.899E-05, *p* *=* 0.0019, *p* *=* 0.0022, *p* *=* 0.0017, **p < 0.01, ***p < 0.001)

Zinc has been reported to induce tyrosine phosphorylation through Src kinases activation^24^. To further explore the upstream mechanism contributing to zinc-induced CDK5-Y15 phosphorylation, N2a cells were pre-incubated with Src kinases inhibitor PP2 before they were subjected to ZnSO_4_ treatment. PP2 almost completely reversed zinc-induced Tyr15 phosphorylation (Fig. 4a) and activation of CDK5 (Fig. 4b). Furthermore, i.c.v. injection of PP2 to rats before they were subjected to unilateral MCAO significantly reduced the brain infarct volume (Fig. 4c) and decreased the hippocampal CA1 neuron death (Fig. 4d). CDK5-Tyr15 phosphorylation was also reversed (Fig. 4e). Laser Doppler flow detection showed that PP2 did not affect the cerebral blood flow (Supplementary Fig 2). We further detected the expression and activity of Src kinases, the results showed that Src was activated (Y416 phosphorylation) in MCAO, whereas PP2 could significantly inhibit Src through increasing phosphorylation of inhibitory Y529 site, reducing phosphorylation of kinase activating site Y416 and decreasing the total Src protein level (Fig. 4f). Thus, zinc induces Tyr15 phosphorylation of CDK5 through Src activation; inhibiting Src prevents CDK5-Tyr15 phosphorylation and CDK5 activating in MCAO, further protects the neurons from ischemic cell death.

### Blockade of CDK5-Tyr15 phosphorylation rescues hippocampal neuronal death in ischemic injury

**Fig. 5:**
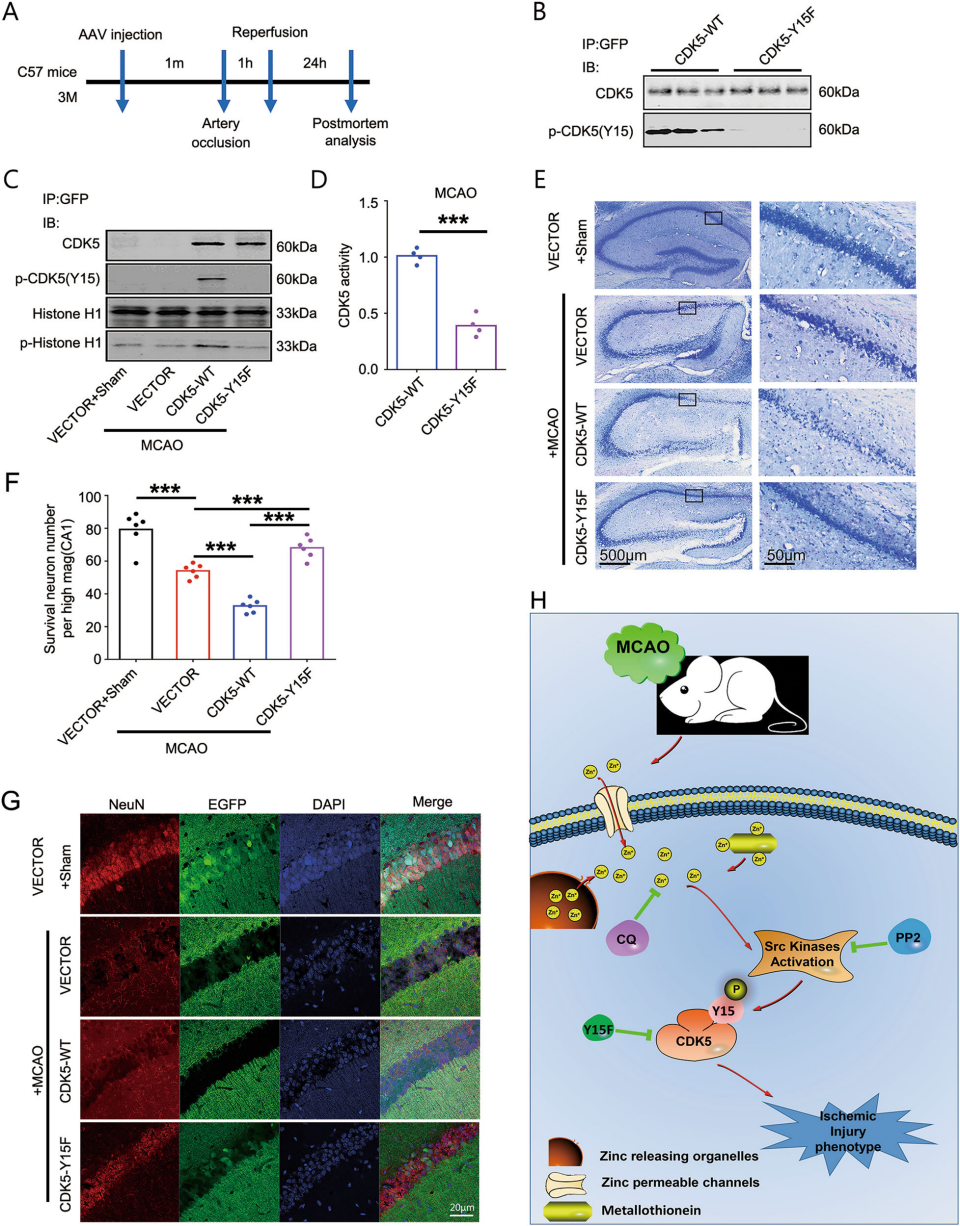
Blockade of CDK5-Tyr15 phosphorylation rescues hippocampal neuronal death in ischemic injury. **a** Timeline of the experiment. **b** Overexpression of Tyr15 unphosphorylable CDK5 in C57BL/6J mice hippocampus. AAV-GFP-CDK5-WT or AAV-GFP-CDK5-Y15F was injected into the hippocampal CA1 region of the mice. Exogenously expressed CDK5 in hippocampus were immunoprecipitated with anti-GFP for western blotting detection of Tyr15-phosphorylated and total CDK5 4 weeks after injection. **c** Blockade of CDK5-Tyr15 phosphorylation by overexpressing GFP-CDK5-Y15F in MCAO mice. GFP-Vector, GFP-CDK5-WT or GFP-CDK5-Y15F overexpressed mice were exposed to MCAO, the hippocampus were homogenized for immnunoprecipitation with anti-GFP. The immnunoprecipitated fractions were divided into two parts, one part for western blotting detection with antibodies against total and Tyr15 phosphorylated CDK5, the other part for CDK5 activity assay. **d** Quantitative analysis of CDK5 activities in **c** (*n* = 4 animals per group, *t*-test, *p* *=* 0.0003, ***p < 0.001). **e-g** Blockade of CDK5-Tyr15 phosphorylation reduced hippocampal neuronal death in MCAO mice. **e** Representative Nissl staining image of the hippocampus sections from Vector + sham, Vector + MCAO, CDK5-WT + MCAO and CDK5-Y15F + MCAO rats. **f** Quantification of survival neuron numbers indicated by Nissl staining in **e** (*n* = 6 animals per group, one-way ANOVA followed by LSD’s post hoc tests, from left to right, *p* *=* 1.62E-05, *p* *=* 4.67E-05, *p* *=* 6.32E-05, *p* *=* 3.12E-07, ***p < 0.001). **g** Representative NeuN immunostaining (red) and GFP (green) image of the hippocampus sections, DAPI staining showed the nuclei. **h** A working model: In ischemic brain injury, [Zn^2+^]_i_ is increased due to extracellular Zn^2+^ influx and intracellular Zn^2+^ release from metallothioneins or organelles (vesicles/mitochondria). Zinc thus activates Src family kinases, and then causes CDK5-Tyr15 phosphorylation and activation, induces ischemic injury phenotype

To validate the hypothesis that Tyr15 phosphorylation by zinc plays a key role in CDK5 activation-induced ischemic injury, GFP-tagged wild-type or Y15F mutated CDK5 were overexpressed in hippocampus of mice through AAV infection 1 month before they were subjected to MCAO (Fig. 5a). The successful expression was confirmed by immunoprecipitating exogenously expressed CDK5 from the hippocampal tissue homogenates with specific anti-GFP antibody (Fig. 5b). CDK5-Y15F mutant failed to be phosphorylated at Tyr15 (Fig. 5c) and activated in mouse hippocampus during MCAO (Fig. 5d). Nissl staining showed that overexpression of wild-type CDK5 resulted in further neuronal loss in hippocampal CA1 compared with vector-expressed mice subjected to MCAO; overexpression of CDK5-Y15F mutant partially rescued the hippocampal CA1 neuronal death (Figs. 5e, f). This result was confirmed by fluorescence staining: MCAO resulted in extensive loss of virus-infected cells (green, Enhanced green fluorescence protein (EGFP) positive) in vector or wild-type CDK5 expressed group, whereas lots of CDK5-Y15F expressed neurons survived after MCAO, which was also confirmed by NeuN (red) staining (Fig. 5g). These data indicate that Tyr15 phosphorylation of CDK5 plays a key role in CDK5 activation and neuronal death in focal cerebral ischemic injury.

## Discussion

Zn^2+^ toxicity plays an important role in ischemic neuronal death. Zn^2+^ accumulation in degenerating neurons was observed in both focal and global models of stroke in vivo^20,25^, and in brain slices exposed to oxygen and glucose deprivation^5,26,27^. [Zn^2+^]_i_ rises preceded [Ca^2+^]_i_ increase and the loss of plasma membrane permeability in ischemic neurons, whereas Zn^2+^ chelation delayed Ca^2+^ deregulation^26^ and prevented cells from ischemic damage^5,20,25,26^. Zn^2+^ is particularly important in causing the selective degeneration of hippocampal CA1 pyramidal neurons. In a transient global ischemia rat model, Zn^2+^, but not Ca^2+^ chelation prevented CA1 neurodegeneration^20^. In another focal ischemia rat model (MCAO), administration of Zn^2+^ chelator reduced the infarct volume significantly in mild focal ischemia, and this protective effect was lost when insult severity was increased^25^. These interesting results indicate that intracellular Zn^2+^ accumulation is an early trigger of the ischemic neurodegenerative cascade.

Aberrant CDK5 activation has been identified to be a principal cause of neuronal death during stroke. Increased CDK5 protein levels or activities were detected in stroke animal models or patients^17,18,28,29^. Correspondingly, pharmacological or genetic inhibition of CDK5 could reduce infarct size, prevent neuronal death and promote functional recovery in animal models^17,30–33^. However, as CDK5 is required in lots of neuronal physiological functions, administration of CDK5 inhibitors are reported to affect physiological functions of CDK5 and lead to serious side effects^34^. Therefore, targeting the upstream factors inducing aberrant CDK5 activation in ischemic brain injury is a therapeutic strategy in stroke patients.

One recognized mechanism underlying CDK5 activation in ischemic injury is calpain-dependent cleavage of p35 to p25, as the cleavage increases the solubility of the CDK5 active complex p25/CDK5 thus enhances it cytoplasmic activity^9^. Increased p25 levels^17,18,30,35^ and calpain activities^36,37^ were observed in ischemic injury. In some animal models and stroke patients, the p35 expression level is also increased^17,38^. On the other side, CDK5 activation by Tyr15 phosphorylation was not investigated in stroke, even though it has been identified in numerous studies related to CDK5 activation in neurite and spine retraction, dendrite outgrowth and Abeta-triggered neurodegeneration^10–12,39^. Given the coincident Zn^2+^ accumulation and CDK5 activation in damaged neurons in stroke, and Zn^2+^ is capable of inducing tyrosine phosphorylation of multiple proteins^24,40–42^, it is of interest to investigate if Zn^2+^ may also modulate CDK5 activity through Tyr15 phosphorylation in ischemic brain injury.

In the present study, we first confirmed the CDK5 activation in ischemic stroke by detecting CDK5 activity in the hippocampal tissues from rat models with MCAO, analysis of the protein levels in the same tissue showed significantly increased Tyr15 phosphorylated CDK5 levels, indicating a possible involvement of Tyr15 phosphorylation in activating CDK5 in this stroke model. The p35 and p25 levels were also increased, which was consistent with previous findings^18,38^. In some stroke models, the increase of p25 is accompanied with decreased but not increased p35 level^17,30^. This difference may depend on the different time point for observation. In a time point when the expression of p35 significantly increased, the increased total p35 level may override the decrease of p35 by cleavage. However, p25 accumulation is a common change in all stroke models, suggesting that p25 contributes to CDK5 activation in ischemic injury. Thus, CDK5 is activated in ischemic stroke and this activation may be caused by CDK5-Tyr15 phosphorylation and/or p25 accumulation.

Next, we explored the effect of chelatable Zn^2+^ in regulating CDK5 in stroke. CQ is a widely used membrane-permeable zinc chelator^22,43^, it is reported that CQ can effectively reduce chelatable zinc in brain^22^ and attenuate the ischemia-induced zinc accumulation in the CA1 pyramidal neurons^23^, thus we used CQ to reveal the effect of zinc on CDK5 activation in focal cerebral ischemia. Pretreatment of CQ efficiently reversed CDK5 activation, reduced infarct volume and prevented hippocampal CA1 neurons from death induced by MCAO, without affecting the cerebral blood flow. At the same time, Tyr15 phosphorylation of CDK5 was also reversed. However, the p35 cleavage to p25 was not reversed. On the contrary, more p35 proteins were cleaved to p25, which was consistent with the calpain activity assay result: MCAO induced calpain activation, whereas CQ pretreatment further increased calpain activity. There are two explanations for this result: (1) Zinc has an inhibitory effect on calpain; (2) CQ directly activate calpain through other unknown mechanism. The first hypothesis was identified by the following observation in our study that Zn^2+^ inhibited calpain in ZnSO_4_-incubated cells. Another zinc chelator TPEN also caused calpain activation in cultured cells^44^. Thus, in our experimental system, Zn^2+^ chelation by CQ induced CDK5 inhibition in rats with MCAO, this effect was not mediated by decreased p35 cleavage, but more possibly by reduced Tyr15 phosphorylation. In another word, Zn^2+^ may cause CDK5 activation through Tyr15 phosphorylation.

To identify this hypothesis, we incubated N2a cells with ZnSO_4_ to reveal the direct effect of zinc on CDK5. Our data showed that Zn^2+^ activated CDK5 through a Tyr15 phosphorylation-dependent manner. Mutation of Tyr15 to non-phosphorable Phe blocked the CDK5 activating by Zn^2+^. Furthermore, Zn^2+^ induced CDK5-Tyr15 phosphorylation through Src kinases activation, as Src kinases inhibitor PP2 completely reversed Zn^2+^-induced Tyr15 phosphorylation and CDK5 activation. The activation of Src kinases by zinc was also identified in our previous study^24^ and by other researchers^41,42^. Thus, zinc activates Src kinases, the latter, causes CDK5-Tyr15 phosphorylation and activation. For a validation of this molecular signaling pathway in ischemic injury, we performed rescue experiments by pretreatment with PP2 or expression of Y15F mutant of CDK5 in hippocampal CA1 regions in MCAO mice. Both strategies could reverse CDK5-Tyr15 phosphorylation and activation, and alleviate the neuronal damage. All these findings support that zinc-induced CDK5-Tyr15 phosphorylation underlies CDK5 activation and promotes ischemic neuronal death in stroke (Fig. 5h).

Our study reveals a new signaling pathway underlying Zn^2+^ toxicity in stroke. [Zn^2+^]_i_ rises is an early event in ischemic neuronal death, based on our data and others, we suspect that the early Zn^2+^ accumulation activates CDK5 through Tyr15 phosphorylation, CDK5 thus promotes [Ca^2+^]_i_ increase by directly phosphorylating NMDA receptors^19^. Subsequently, calpain is activated and induces p25-dependent CDK5 activation. The persistent CDK5 activation causes neuronal death. This may explain that zinc chelation showed the best prevention in transient and mild ischemic models, but not in severe cases^25^. As in severe ischemic injury, multifaceted and complex cascades such as glutamate excitotoxicity and oxidative stress are fully motivated as the executors of neuronal death. Thus, an early intervention of [Zn^2+^]_i_ rises and CDK5-Tyr15 phosphorylation is a promising therapeutic strategy in the treatment of stroke. Zn^2+^ chelators have the potential to be used in the patients treated with intravenous thrombolysis (within 3 h of ischemic stroke onset)^45^ as an adjunctive therapy; or in the patients treated with mechanical thrombectomy through intra-arterial delivery to prevent the reperfusion injury. Its application as a neuroprotective intervention in stroke is worthy of further exploration and identification.

## Electronic supplementary material

Supplementary Figure 1

Supplementary Figure 2

supplementary information
