# Supplementary material for: Zinc induces CDK5 activation and neuronal death through CDK5-Tyr15 phosphorylation in ischemic stroke
Source: Cell Death Dis. 2018 Aug 29;9(9):870. doi: 10.1038/s41419-018-0929-7 (PMC6115431; doi:10.1038/s41419-018-0929-7)
Supplement: Supplementary file 3 — supplementary information [file 41419_2018_929_MOESM3_ESM.docx]

**

**

**Supplementary Figure 1. The real-time blood perfusion images show no differences between MCAO and CQ+MCAO rats. *A,*** Representative images obtained from PeriCam PSI System. The brighter area indicates higher blood perfusion. ***B,*** Mean impact of occlusion and reperfusion upon blood flow in 3-month-old rats. n = 3 animals per group.

**

**

**Supplementary Figure 2. The real-time blood perfusion images show no differences between MCAO and PP2+MCAO rats. *A,*** Representative images obtained from PeriCam PSI System. The brighter area indicates higher blood perfusion. ***B,*** Mean impact of occlusion and reperfusion upon blood flow in 3-month-old rats. n = 3 animals per group.
